# The clinical and pathological features of low-grade epilepsy-associated glioneuronal tumors

**DOI:** 10.1038/s41598-022-22443-2

**Published:** 2022-10-28

**Authors:** Mingguo Xie, Xiongfei Wang, Jiao Qiao, Jian Zhou, Yuguang Guan, Tianfu Li, Xueling Qi, Guoming Luan

**Affiliations:** 1 Department of Neurosurgery, Epilepsy Center, Sanbo Brain Hospital, Capital Medical University, Xiangshan Yikesong Road 50, Haidian District, Beijing, 100093 China; 2 Beijing Key Laboratory of Epilepsy, Sanbo Brain Hospital, Capital Medical University, Beijing, China; 3 Beijing Institute for Brain Disorders, Capital Medical University, Beijing, China; 4 Department of Neurology, Epilepsy Center, Sanbo Brain Hospital, Capital Medical University, Beijing, China; 5 Department of Neuropathology, Epilepsy Center, Sanbo Brain Hospital, Capital Medical University, Beijing, China

**Keywords:** Neuroscience, Biomarkers, Molecular medicine, Oncology

## Abstract

The aim of the study was to evaluate the clinicopathological features, as well as the surgical prognosis, of epilepsy-associated glioneuronal tumors (GNT) with CD34 expression and BRAF mutation. Clinical data of patients who underwent epilepsy surgery for GNT were retrospectively studied. Univariate and multivariate analyses were performed to evaluate the correlations of clinical and pathological factors with molecular markers of CD34 expression and BRAF^V600E^ mutation in GNT. A total of 247 patients with GNT had immunohistochemical detection of CD34 expression (CD34 positive vs. negative: 198/49), and among them, 102 patients had immunohistochemical detection of BRAF^V600E^ mutation (BRAF positive vs. negative: 59/43). Univariate analysis found that tumor types (P < 0.001), patient population (P = 0.015), seizure aura (P = 0.007), drug-resistant epilepsy (P = 0.036), concordance of ictal electroencephalogram (EEG) findings (P = 0.032), surgical resection extent (P = 0.045), tumor location (P = 0.007) and duration of epilepsy (P = 0.027) were related to CD34 expression, and that concordance of ictal EEG findings (P = 0.031) and age at surgery (P = 0.015) were related to BRAF^V600E^ mutation. In addition, history of generalized tonic–clonic seizure (HR 0.12; P = 0.035), drug-resistant epilepsy (HR 0.13; P = 0.030) and concordance of interictal EEG findings (HR 8.01; P = 0.039) were associated with tumor progression-free survival (PFS). However, CD34 expression or BRAF^V600E^ mutation in GNT was not associated with surgical outcomes of seizure control and tumor PFS. The CD34 expression or BRAF^V600E^ mutation in GNT may partly influence the distribution of clinicopathological features of patients with epilepsy, but they may be not able to predict the surgical prognosis of seizure outcome and tumor recurrence.

## Introduction

Brain tumors are frequently met in patients with epilepsy surgery, and the glioneuronal tumors (GNT) are the most common brain tumors accompanied by seizures, which primarily comprise ganglioglioma (GG) and dysembroplastic neuroepithelial tumor (DNT)^1,2^. Recently, the molecular markers of CD34 and BRAF mutation were interestingly found to be associated with brain tumors with epilepsy, especially GNT^3–7^.

CD34 was first identified on hematopoietic progenitor cells as a transmembrane phosphoglycoprotein that seems to play a role in cell adhesion, differentiation and proliferation^8^. CD34 expression is also found on several nonhematopoietic cell types, such as vascular endothelial progenitors, embryonic fibroblasts and epithelial progenitors, thus being regarded as a general marker of progenitor cells^8,9^. Recently, CD34 has been found transiently expressed in the central nervous system during early neurulation but not in mature neuroectodermal cell progenies in the normal brain^5^. Although CD34-positive cells have been reported in gliosarcoma and giant cell variant of glioblastoma, or non-neoplastic glioneuronal hamartias or hamartomas, they are particularly represented in low-grade or developmental brain tumors, such as GNT (GG/DNT), pleomorphic xanthoastrocytoma (PXA) and pilocytic astrocytoma (PA), and so on, all of which are interestingly associated with chronic epilepsy^3–5,10^.

BRAF (v-raf murine sarcoma viral oncogene homolog B1) is a member of the RAF family of serine/threonine protein kinases, playing a critical role in transducing signals from membrane-bound, GTP-loaded RAS proteins to MEK and ERK kinases (RAS/RAF/MEK/ERK pathway)^11,12^. The vast majority of BRAF mutations (> 90%) affect a mutational hot spot at amino acid position 600 and are characterized by the exchange of Valine by Glutamate, thus referred to as BRAF Val600Glu (or BRAF^V600E^), which generates a constitutively active monomeric protein with high kinase activity that does not require RAS signaling^12,13^. BRAF is mutated in about 8% of all human cancers^13^. These mutations primarily occur in melanomas and at much lower frequency in thyroid, lung, and colorectal cancer^11,13^. Recently, BRAF^V600E^ mutations are found in tumors of the central nervous system, mainly affecting glial or glioneuronal tumors that include PXA, GG, DNT and PA, as well as pediatric astrocytoma^6,7,13^, but glioblastoma and other gliomas were with low frequency or absence of mutations, and no mutations were detected in non-glial tumors, such as embryonal tumors, meningiomas, nerve sheath tumors and pituitary adenomas^6,7^. More recently, the monoclonal BRAF^V600E^ mutation-specific antibody via immunohistochemistry (IHC) detection has been found and widely used to screen for BRAF^V600E^ mutation in the diagnostic work-up in place of directly genetic sequencing^1,14^, since the first BRAF^V600E^ specific antibody was reported in 2011 (clone VE1)^15^.

Although the molecular expression of CD34 and BRAF^V600E^ mutation could frequently and exclusively occur in GNT with epilepsy, their clinicopathological features were not yet well defined, as well as the prediction of long-term seizure outcome and tumor recurrence or progression^3,5,6,16,17^. Thus, we particularly reported our surgical series of GNT further to evaluate the associations of clinicopathological features, as well as surgical prognosis, with molecular expression of CD34 and BRAF^V600E^ mutations in GNT with epilepsy.

## Methods

### Patient selection

A retrospective chart review was conducted for all patients with epilepsy who underwent surgical treatment for GNT between 2008 and 2021 at Sanbo Brain Hospital, Capital Medical University. This study was approved by the Capital Medical University Sanbo Brain Hospital Ethics Committee, with the consent waiver obtained due to there no personally identifiable data in the study, and all methods were performed in accordance with relevant guidelines and regulations.

Patients who had epilepsy caused by brain tumors that were histopathologically confirmed as GG or DNT were enrolled in the study. The clinical data of patients with tumors that were detected by IHC with molecular markers of CD34 and BRAF^V600E^ mutation was analyzed. Finally, a total of 247 consecutive patients who had tumors with IHC examination of CD34 expression, including 102 patients with IHC examination of BRAF^V600E^ mutation, were enrolled in the study.

### Preoperative evaluation and surgery

Individualized preoperative evaluations, including detailed medical history taking, seizure semiology, video electroencephalogram (EEG) and brain magnetic resonance imaging (MRI), were performed for each patient. The video EEG monitoring was performed in all patients (at least 16 h), and the concordant EEG findings of interictal or ictal epileptiform discharges (EDs) were defined as EDs sources localized in the same tumor-invading brain hemisphere. In addition, the lesion size was calculated by the mean tumor diameter on T1-weighted MRI scans.

Surgical plans were made by a multidisciplinary meeting based on detailed preoperative evaluations. The aim of the operation was to remove the tumor as well as the relevant epileptogenic zone (EZ), which was determined by the findings of preoperative evaluations and/or intraoperative electrocorticography (ECoG). When operating, neurological electrophysiological monitoring and neuronavigation were performed for the sake of safe tumor resection. In particular, according to the resection extent of brain tissue based on comparison of preoperative and postoperative MRI, extensive tumor resection was defined as resection of both tumor and peritumoral cortex (or hippocampus), or otherwise the simple tumor resection.

### Immunohistochemical staining and pathological diagnosis

The surgically removed brain tissue specimens were fixed with 3.7% neutral formaldehyde, embedded in conventional paraffin, sliced into 5-μm thick sections, and then stained with hematoxylin and eosin (H&E). Immunohistochemical staining was performed with the following primary antibodies: anti-BRAF^V600E^, glial fibrillary acidic protein (GFAP), neuronal nuclear antigen (NeuN), neurofilament (NF), synaptophysin, oligodendrocyte transcription factor 2 (Olig-2), epithelial cell membrane antigen (EMA), Ki-67, p53, CD68, CD34, isocitrate dehydrogenase (IDH1/2).

Histopathological records were systematically reviewed by two experienced neuropathologists according to the WHO classification scheme from 2016, including a panel of immunohistochemical markers. In particular, tumors with IHC detection of CD34 expression and BRAF^V600E^ mutation were analyzed in terms of their associations with clinical and pathological features.

### Follow-up examination

Patients were periodically followed up at the 3rd month and 6th month postoperatively and yearly thereafter. Follow-up evaluations of seizure and tumor recurrence or progression, as well as neurological statuses, were performed by neurosurgeons at the clinic and/or by telephone interview in each patient. Favorable seizure outcomes with Engel classification were defined as Engel class I, and unfavorable seizure outcomes were Engel class II–IV at the last follow-up evaluation.

### Study variables and statistical analysis

Clinical variables of interest were compared between two groups of patients with or without CD34 expression and BRAF^V600E^ mutation, which included patient or demographic characteristics, tumor characteristics, seizure semiology and electrophysiological findings, as well as operative and follow-up variables. Among them, categorical variables were described with absolute value and percentages, while continuous variables were described with medians and interquartile ranges (IQR).

Descriptive statistics between compared groups were tested by t tests and χ^2^ tests. When necessary, the Kruskal–Wallis rank-sum test and the Fisher’s exact test were used for continuous and categorical variables, respectively. Variables with a P value < 0.05 in the univariate analysis were then entered into the multivariate binary logistic or Cox regression model. Odds ratios (OR) and hazard ratios (HR) were presented with 95% confidence intervals (CI). Statistical tests were considered significant if P value < 0.05. All data were analyzed by the software package SPSS, version 21.

### Ethics approval

The Research Ethics Committee of Sanbo Brain Hospital approved the study. The manuscript does not contain individual clinical data, and informed consent was not required.

## Results

### Patient demographics

Of the 247 patients, 93 patients (37.7%%) were female and 108 patients (43.7%) were children (age < 18 years old). The median age at surgery was 20 years (IQR 10.5–26 years), the median age of seizure onset was 10 years (IQR: 4–17 years), and the median duration of epilepsy was 60 months (IQR 12–144 months) (Table 1). Upon admission, 185 patients (74.9%) were with drug-resistant epilepsy.

**Table 1:** Univariate analysis of the relationships between CD34 expression in GNT and clinicopathology in 247 patients.

Variable	Subtype	CD34 expression		In total	P value
		CD34 (−)	CD34 (+)		
BRAF^V600E^ mutation, n (%)	BRAF (−)	8 (18.6%)	35 (81.4%)	43	0.074
	BRAF (+)	6 (10.2%)	53 (89.8%)	59	
	Unknown^a^	35 (24.1%)	110 (75.9%)	145	
Tumor type, n (%)	DNT	24 (63.2%)	14 (36.8%)	38	0.000^c^
	GG	24 (12.1%)	174 (87.9%)	198	
	Mixed GNT	1 (9.1%)	10 (90.9%)	11	
Patient gender, n (%)	Male	30 (19.5%)	124 (80.5%)	154	0.856
	Female	19 (20.4%)	74 (79.6%)	93	
Patient population, n (%)	Children	29 (26.9%)	79 (73.1%)	108	0.015^c^
	Adult	20 (14.4%)	119 (85.6%)	139	
Drug-resistant epilepsy, n (%)	No	18 (29%)	44 (71%)	62	0.036^c^
	Yes	31 (16.8%)	154 (83.2%)	185	
Seizure type, n (%)	Focal	31 (18.7%)	135 (81.3%)	166	0.512
	Generalized	18 (22.2%)	63 (77.8%)	81	
Seizure aura, n (%)	No	32 (26.9%)	87 (73.1%)	119	0.007^c^
	Yes	17 (13.3%)	111 (86.7%)	128	
History of GTCS, n (%)	No	19 (18.3%)	85 (81.7%)	104	0.598
	Yes	30 (21%)	113 (79%)	143	
History of SE, n (%)	No	47 (19.6%)	193 (80.4%)	240	0.915
	Yes	2 (28.6%)	5 (71.4%)	7	
Seizure frequency, n (%)	Daily	17 (23%)	57 (77%)	74	0.171
	Weekly	12 (13.5%)	77 (86.5%)	89	
	Monthly	11 (20%)	44 (80%)	55	
	Quarterly or yearly	9 (31%)	20 (69%)	29	
Concordant interictal EEG findings, n (%)	No	13 (25.5%)	38 (74.5%)	51	0.058
	Yes	27 (16%)	142 (84%)	169	
	Unknown^b^	9 (33.3%)	18 (66.7%)	27	
Concordant ictal EEG findings, n (%)	No	13 (26%)	37 (74%)	50	0.032^c^
	Yes	15 (12.8%)	102 (87.2%)	117	
	Unknown^b^	21 (26.3%)	59 (73.8%)	80	
Resection extent, n (%)	Simple tumor resection	26 (26%)	74 (74%)	100	0.045^c^
	Extensive tumor resection	23 (15.6%)	124 (84.4%)	147	
Tumor side, n (%)	Right	23 (17.8%)	106 (82.2%)	129	0.408
	Left	26 (22%)	92 (78%)	118	
Tumor location, n (%)	Multilobe	6 (23.1%)	20 (76.9%)	26	0.007^c^
	Temporal	24 (14.5%)	141 (85.5%)	165	
	Non-temporal	19 (33.9%)	37 (66.1%)	56	
Tumor calcification, n (%)	No	37 (21.5%)	135 (78.5%)	172	0.318
	Yes	12 (16%)	63 (84%)	75	
Tumor encystation, n (%)	No	40 (19.5%)	165 (80.5%)	205	0.777
	Yes	9 (21.4%)	33 (78.6%)	42	
Ki67 index, n (%)	0–1%	29 (17.5%)	137 (82.5%)	166	0.381
	2–5%	19 (24.4%)	59 (75.6%)	78	
	> 5%	1 (33.3%)	2 (66.7%)	3	
Tumor-associated FCD, n (%)	No	34 (18.8%)	147 (81.2%)	181	0.492
	Yes	15 (22.7%)	51 (77.3%)	66	
Concomitant HS, n (%)	No	46 (20.2%)	182 (79.8%)	228	0.872
	Yes	3 (15.8%)	16 (84.2%)	19	
Seizure outcome, n (%)	Engel class I	38 (19.4%)	158 (80.6%)	196	0.807
	Engel class II–IV	7 (21.2%)	26 (78.8%)	33	
Tumor recurrence, n (%)	No	49 (20.3%)	192 (79.7%)	241	0.474
	Yes	0 (0%)	6 (100%)	6	
Age at surgery, median (IQR)	In years	14 (7.3–26)	20 (12.4–26)	20 (10.5–26)	0.064
Age of seizure onset, median (IQR)	In years	8.5 (3.3–18)	10.3 (4.4–16.6)	10 (4–17)	0.403
Duration of epilepsy, median (IQR)	In months	36 (10–108)	60 (18–156)	60 (12–144)	0.027^c^
Tumor size, median (IQR)	In millimeter	20 (15–22.5)	17.5 (15–20)	17.5 (15–20)	0.145
Hospitalization time, median (IQR)	In days	23 (17.5–32)	25 (19–30)	24 (18–30)	0.912
Follow-up time, median (IQR)	In months	60 (31.5–84.5)	53 (25.7–76.3)	54 (26–78)	0.194

*GG* ganglioglioma, *DNT* dysembryoplastic neuroepithelial tumor, *GNT* glioneuronal tumor, *GTCS* generalized tonic–clonic seizure, *SE* status epilepticus, *EEG* electroencephalogram, *FCD* focal cortical dysplasia, *HS* hippocampus sclerosis, *IQR* interquartile range.

^a^The unknown cases were those without detection of BRAF^V600E^ mutation.

^b^Patients with unknown results in lateral concordant EEG findings of interictal epileptiform discharges and of ictal seizure rhythms were recorded in 27 cases (no IEDs or normal EEG findings) and 80 cases (no ictus during video EEG monitoring), respectively.

^c^P < 0.05, with significance.

### Tumor characteristics

Of the 247 tumors found by MRI, 118 cases (47.8%) were in the left brain. In particular, 165 patients (66.8%) had tumors located in the temporal lobe. Tumors located in the frontal, parietal, occipital, insular and multiple lobes were found in 28 (11.2%), 15 (6%), 10 (4%), 3 (1.2%) and 26 (10.4%) cases, respectively. The median tumor size was 17.5 mm (IQR 15–20 mm) (Table 1).

According to postoperative pathological records of surgical specimens, all 247 lesions were diagnosed as low-grade glioneuronal tumors, including GG (198), DNT (38), and GNT with mixed characteristics of GG and DNT, PXA or astrocytoma (mixed GNT, 11). Tumor-associated focal cortical dysplasia (FCD) was recorded in 66 patients (26.7%), including 28 cases (11.3%) of FCD II. Concomitant hippocampus sclerosis was found in 19 patients (7.7%). Tumors with tissue calcification and encystation were recorded in 75 cases (30.4%) and 42 cases (17%), respectively. The Ki67 index of tumor tissue was categorized into three subgroups: 0–1% (166 cases), 2–5% (78 cases) and 6–12% (3 cases). In particular, the IHC detection of CD34 positive expression was found in 198 (80.2%) patients, while CD34 negative expression was found in 49 patients (19.8%). Of the 102 patients (41.3%) with IHC detection of BRAF^V600E^ mutation, 59 cases (57.8%) were BRAF positive (Table 1). In addition, 214 cases (86.6%) were tested with IDH mutations, but no IDH (+) was found in all tested lesions of GG (171), DNT (32) and mixed GNT (11).

### Seizure semiology and electrophysiological findings

Before surgery, 74 patients (30%) complained of daily seizure onsets, while the other 173 patients (70%) experienced seizure onsets weekly (85), monthly (59), quarterly or yearly (29). A total of 166 patients (67.2%) had focal seizures as the most common seizure onset in recent years, while 81 patients (37.8%) had generalized seizures. In addition, history of seizure auras, generalized tonic–clonic seizures (GTCS) and status epilepticus (SE) were recorded in 128 (51.8%), 143 (57.9%) and 7 (2.8%) patients, respectively.

Regarding video EEG findings, concordant interictal EEG findings were found in 169 patients (68.4%), while discordant findings were in 51 patients (20.6%); 27 patients (10.9%) were with unknown results due to lack of significant epileptiform discharges or being in a normal EEG setting. Concordant EEG findings of ictal seizure rhythms were found in 117 patients (47.4%), and discordant findings were found in 50 patients (20.2%), but 80 patients (32.4%) were with unknown results due to no ictal seizures (Table 1).

### Surgical results

Intraoperative ECoG monitoring was performed in 194 patients (78.5%). Complete tumor resection was achieved in 245 patients (99.2%), and 2 cases were with subtotal tumor resection because of tumors invading brain eloquent areas. In total, extensive tumor resection was performed in 147 patients (59.5%), and simple tumor resection was in 100 patients (40.5%).

Postoperatively, 33 patients (13.4%) had acute seizures within the first 2 weeks after surgery. Operation-associated complications were met in 33 patients (13.4%), including venous thrombosis (2), pulmonary infection (3), intracranial infection (8), hemorrhagic apoplexy (3), cerebral infarction (4), incision infection or poor healing (6), and others (8; such as electrolyte disorders, urinary tract infection and gastrointestinal dysfunction). New neurological deficits were recorded in 28 patients (11.3%), including, muscle weakness (14), impaired vision (9), aphasia (4), decreasing memory (4), mental disorder (4), eyelid drooping (3) and facial paralysis (1). The median time of hospitalization was 24 days (IQR 18–30 days).

### Follow-up outcomes

All patients were followed up, except for 14 patients (5.7%) lost, with the median follow-up time of 54 months (IQR 26–78 months). Of 229 patients who were followed up for at least 12 months, 196 patients (85.6%) were seizure-free and had a favorable seizure outcome (Engel class I), while 33 patients (14.4%) had an unfavorable seizure outcome (Engel class II/12, III/14 and IV/7). In total, 161 patients (70.3%) had anti-epileptic drugs reduced (45) or discontinued (116). During the whole follow-up period, 6 (2.6%) patients had tumor recurrence, including one with subtotal tumor resection, and the 10-year tumor progression-free survival (PFS) was 95%. Among them, 3/6 of cases had seizure recurrence, and 2 cases of GG had malignant progression (one also with seizure recurrence).

### Univariate and multivariate analyses

Clinical and pathological factors in 247 patients were compared between two groups [tumor with CD34 (+) vs. CD34 (−)] (Table 1). Significant differences were found in tumor types (GG vs. DNT, P < 0.001), patient population (children vs. adults, P = 0.015), seizure aura (P = 0.007), drug-resistant epilepsy (P = 0.036), concordance of ictal EEG findings (concordant vs. discordant, P = 0.032), surgical resection extent (simple tumor resection vs. extensive tumor resection, P = 0.045), tumor location (temporal vs. non-temporal, P = 0.007) and duration of epilepsy (P = 0.027). In particular, surgical outcomes of seizure control (P = 0.807) and tumor recurrence (P = 0.474) were not found with differences between two groups. Multivariate binary logistic regression analysis finally included the tumor type (GG vs. DNT, P < 0.001; OR 13.3, 95% CI 5.9–29.9) and the patient population (adults vs. children, P = 0.014; OR = 2.5, 95% CI 1.2–5.2) into the predicting model of GNT with CD34 positive expression.

The clinical and pathological features of 102 patients who had IHC detection of BRAF^V600E^ mutation were also compared between two groups [tumor with BRAF (+) vs. BRAF (−)] (Table 2). Significant differences were found in concordance of ictal EEG findings (concordant vs. discordant, P = 0.031) and age at surgery (P = 0.015), but not in surgical outcomes of seizure control (P = 0.937) and tumor recurrence (P = 1.000). Finally, only the age at surgery (P = 0.019, OR 1.05, 95% CI 1.01–1.10) was found with significance in multivariate binary logistic regression model.

**Table 2:** Univariate analysis of the relationships between BRAF^V600E^ mutation in GNT and clinicopathology in 102 patients.

Variables	Subtype	BRAF^V600E^ mutation		In total	p value
		BRAF (−)	BRAF (+)		
CD34 expression, n (%)	CD34 (−)	8 (57.1%)	6 (42.9%)	14	0.222
	CD34 (+)	35 (39.8%)	53 (60.2%)	88	
Tumor type, n (%)	DNT	8 (61.5%)	5 (38.5%)	13	0.273
	GG	32 (38.6%)	51 (61.4%)	83	
	Mixed GNT	3 (50%)	3 (50%)	6	
Patient gender, n (%)	Male	24 (39.3%)	37 (60.7%)	61	0.483
	Female	19 (46.3%)	22 (53.7%)	41	
Patient population, n (%)	Children	23 (50%)	23 (50%)	46	0.146
	Adult	20 (35.7%)	36 (64.3%)	56	
Drug-resistant epilepsy, n (%)	No	17 (53.1%)	15 (46.9%)	32	0.129
	Yes	26 (37.1%)	44 (62.9%)	70	
Seizure type, n (%)	Focal	27 (42.9%)	36 (57.1%)	63	0.856
	Generalized	16 (41%)	23 (59%)	39	
Seizure aura, n (%)	No	19 (38.8%)	30 (61.2%)	49	0.506
	Yes	24 (45.3%)	29 (54.7%)	53	
History of GTCS, n (%)	No	22 (42.3%)	30 (57.7%)	52	0.975
	Yes	21 (42%)	29 (58%)	50	
History of SE, n (%)	No	43 (43%)	57 (57%)	100	0.620
	Yes	0 (0%)	2 (100%)	2	
Seizure frequency, n (%)	Daily	13 (40.6%)	19 (59.4%)	32	0.821
	Weekly	14 (37.8%)	23 (62.2%)	37	
	Monthly	11 (50%)	11 (50%)	22	
	Quarterly or yearly	5 (45.5%)	6 (54.5%)	11	
Concordant interictal EEG findings, n (%)	No	8 (30.8%)	18 (69.2%)	26	0.329
	Yes	30 (47.6%)	33 (52.4%)	63	
	Unknown^a^	5 (38.5%)	8 (61.5%)	13	
Concordant ictal EEG findings, n (%)	No	4 (21.1%)	15 (78.9%)	19	0.031^b^
	Yes	23 (50%)	23 (50%)	46	
	Unknown^a^	16 (43.2%)	21 (56.8%)	37	
Resection extent, n (%)	Simple tumor resection	19 (44.2%)	24 (55.8%)	43	0.606
	Extensive tumor resection	24 (40.7%)	35 (59.3%)	56	
Tumor side, n (%)	Right	22 (41.5%)	31 (58.5%)	53	0.890
	Left	21 (42.9%)	28 (57.1%)	49	
Tumor location, n (%)	Temporal	27 (38%)	44 (62%)	71	0.294
	Non-temporal	19 (57.1%)	9 (42.9%)	21	
	Multilobe	4 (40%)	6 (60%)	10	
Tumor calcification, n (%)	No	24 (37.5%)	40 (62.5%)	64	0.216
	Yes	19 (50%)	19 (50%)	38	
Tumor encystation, n (%)	No	35 (41.2%)	50 (58.8%)	85	0.654
	Yes	8 (47.1%)	9 (52.9%)	17	
Ki67 index, n (%)	0–1%	25 (41.7%)	35 (58.3%)	60	0.682
	2–5%	16 (41%)	23 (59%)	39	
	> 5%	2 (66.7%)	1 (33.3%)	3	
Tumor-associated FCD, n (%)	No	39 (41.5%)	55 (58.5%)	94	0.924
	Yes	4 (50%)	4 (50%)	8	
Concomitant HS, n (%)	No	41 (43.6%)	53 (56.4%)	94	0.515
	Yes	2 (25%)	6 (75%)	8	
Seizure outcomes, n (%)	Engel class I	34 (40.5%)	50 (59.5%)	84	0.937
	Engel class II–IV	5 (41.7%)	7 (58.3%)	12	
Tumor recurrence, n (%)	No	43 (42.6%)	58 (57.4%)	101	1.000
	Yes	0 (0%)	1 (100%)	1	
Age at surgery, median (IQR)	In years	15 (7–23)	20 (14–27)	19 (10.5–25.3)	0.015^b^
Age of seizure onset, median (IQR)	In years	7 (4–15)	12 (5.5–18.5)	10 (5–18)	0.106
Duration of epilepsy, median (IQR)	In months	24 (5–108)	42 (12–180)	36 (11.5–126)	0.095
Tumor size, median (IQR)	In millimeter	20 (15–20)	17.5 (15–25)	17.5 (15–21.3)	0.217
Hospitalization time, median (IQR)	In days	20 (16–26)	24 (16–30)	22 (16–28.3)	0.188
Follow-up time, median (IQR)	In months	43 (25–52)	28 (16–52)	37 (21.7–52)	0.312

*GG* ganglioglioma, *DNT* dysembryoplastic neuroepithelial tumor, *GNT* glioneuronal tumor, *GTCS* generalized tonic–clonic seizure, *SE* status epilepticus, *EEG* electroencephalogram, *FCD* focal cortical dysplasia, *HS* hippocampus sclerosis, *IQR* interquartile range.

^a^Patients with unknown results in lateral concordant EEG findings of interictal epileptiform discharges and of ictal seizure rhythms were recorded in 13 cases (no IEDs or normal EEG findings) and 37 cases (no ictus during video EEG monitoring), respectively.

^b^P < 0.05, with significance.

### Kaplan Meier curve and Cox regression analysis

**Figure 1:**
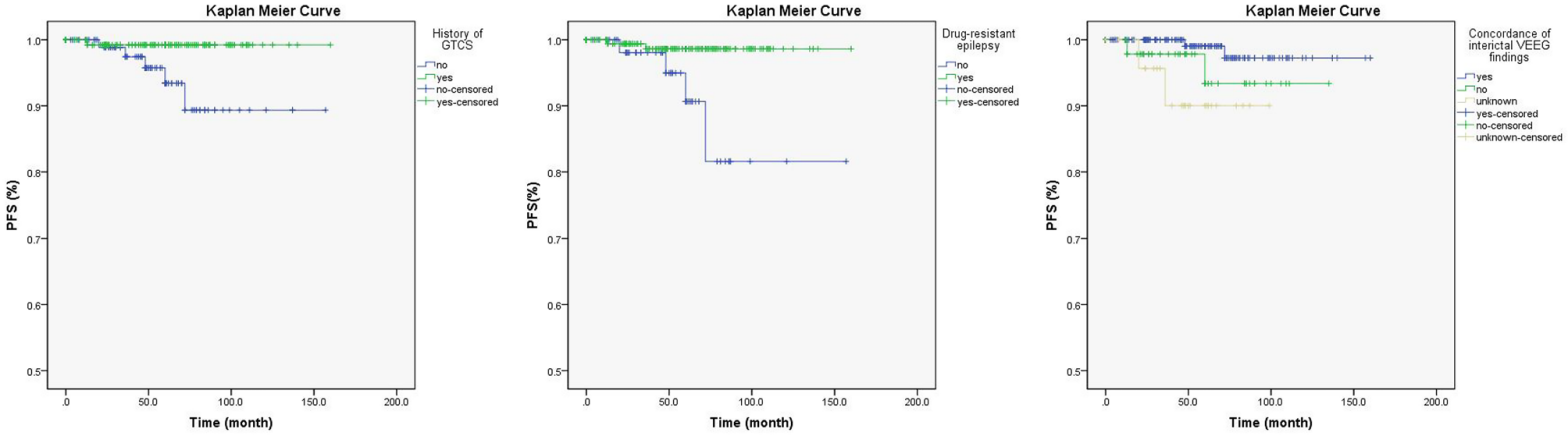
The comparison of Kaplan Meier curves of significant clinical factors in univariate Cox regression analysis, including drug-resistant epilepsy (left), history of generalized tonic–clonic seizure (GTCS; middle) and concordance of interictal video electroencephalogram (VEEG) findings (right).

**Figure 2:**
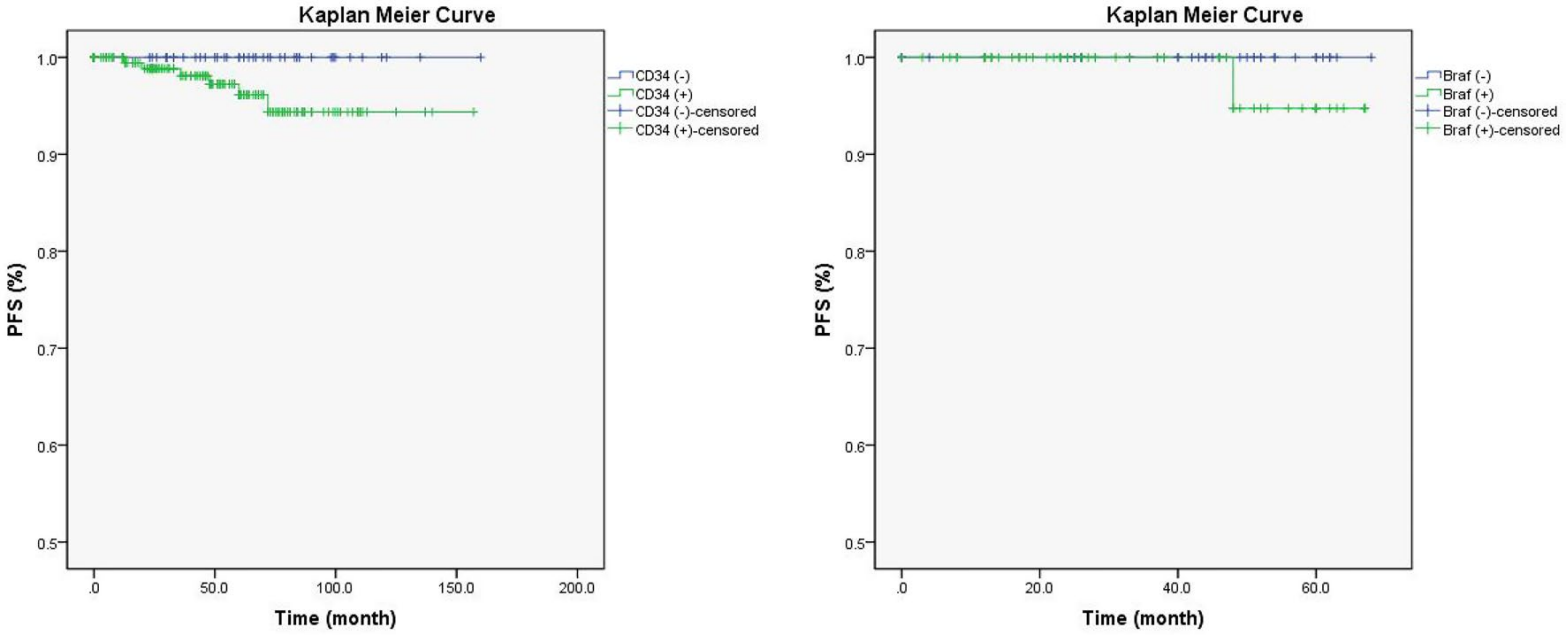
The comparison of Kaplan–Meier curves of CD34 expression and BRAF^V600E^ mutation in glioneuronal tumors with epilepsy.

Univariate Cox regression analysis found the history of GTCS (HR 0.12, P = 0.035), drug-resistant epilepsy (HR 0.13, P = 0.030) and concordant interictal EEG findings (unknown vs. concordant; HR 8.01, P = 0.039) were associated with longer PFS (Table 3, Fig. 1), but only the drug-resistant epilepsy (P = 0.030) was with significance in the multivariate Cox regression analysis. In particular, when compared the Kaplan Meier curves between groups [tumor with CD34 (+) vs. CD34 (−)] or groups [tumor with BRAF (+) vs. BRAF (−)], no difference was found in patients with detection of CD34 expression (χ^2^ = 1.662, P = 0.192) or in patients with detection BRAF^V600E^ mutation (χ^2^ = 0.842, P = 0.359) (Fig. 2).

**Table 3:** Univariate Cox regression analysis of the associations of clinical factors with tumor progression-free survival.

Variable	*B*	HR (95.0% CI)	*P* value
CD34 expression	3.38	29.51 (0.01–57.54)	0.426
BRAF^V600E^ mutation	4.02	55.62 (0.1–108.46)	0.626
Patient gender (female vs. male)	− 1.25	0.29 (0.03–2.45)	0.253
Patient population (adult vs. children)	0.48	1.62 (0.30–8.86)	0.578
Age at surgery, in years	0.02	1.02 (0.96–1.08)	0.563
Age of seizure onset, in years	0.04	1.04 (0.97–1.11)	0.292
Duration of epilepsy, in months	− 0.01	0.99 (0.98–1.01)	0.473
Drug-resistant epilepsy	− 2.04	0.13 (0.02–0.72)	0.030^b^
Seizure type (generalized vs. focal)	− 0.88	0.42 (0.05–3.55)	0.422
Seizure aura	− 1.68	0.19 (0.02–1.60)	0.126
History of GTCS	− 2.08	0.12 (0.01–1.07)	0.035^b^
History of SE	− 3.04	0.05 (0.01–0.10)	0.789
Seizure frequency (monthly vs. non-monthly)	− 0.55	0.57 (0.07–4.94)	0.614
Interictal EEG findings (discordant vs. concordant)	1.29	3.64 (0.51–25.89)	0.196
Interictal EEG findings (unknown vs. concordant)^a^	2.08	8.01 (1.12–57.51)	0.039^b^
Ictal EEG findings (discordant vs. concordant)	0.73	2.07 (0.29–14.70)	0.468
Ictal EEG findings (unknown vs. concordant)^a^	0.48	1.61 (0.23–11.49)	0.632
Tumor type (GG vs. DNT)	0.06	1.06 (0.12–9.06)	0.960
Tumor size, in millimeter	0.08	1.08 (1.00–1.17)	0.059
Tumor side (left vs. right)	0.05	1.05 (0.21–5.21)	0.952
Temporal invasion (temporal vs. non-temporal)	0.74	2.1 (0.25–17.98)	0.498
Tumor calcification	0.31	1.36 (0.25–7.44)	0.726
Tumor encystation	1.09	2.97 (0.54–16.48)	0.212
Ki67 index (2–5% vs. ≤ 1%)	0.92	2.5 (0.58–10.82)	0.220
Tumor-associated FCD	− 1.02	0.36 (0.04–3.11)	0.353
Concomitant HS	− 3.09	0.05 (0.01–0.10)	0.689
Resection extent (extensive tumor resection vs. simple tumor resection)	0.09	1.10 (0.47–2.57)	0.833
Hospitalization time, in days	− 0.05	0.95 (0.86–1.06)	0.358
New neurological deficit	− 0.12	0.88 (0.16–4.87)	0.888
Seizure outcome (unfavorable vs. favorable)	0.17	1.18 (0.14–10.17)	0.878
Follow-up time, in months	0.01	1.01 (0.98–1.04)	0.422

*GTCS* generalized tonic–clonic seizure, *SE* status epilepticus, *EEG* electroencephalogram, *GG* ganglioglioma, *DNT* dysembryoplastic neuroepithelial tumor, *FCD* focal cortical dysplasia, *HS* hippocampus sclerosis, *HR* hazard ratio, *CI* confidence interval.

^a^Patients with unknown results in lateral concordant EEG findings of interictal epileptiform discharges and of ictal seizure rhythms were those with no IEDs or normal EEG findings and those with no ictus during video EEG monitoring.

^b^P < 0.05, with significance.

## Discussion

The molecular markers of CD34 and BRAF^V600E^ mutation are frequently met in GNT^4–7^. Although a few of pediatric gliomas share these molecular features with GNT^3,5,10^, CD34 expression and BRAF^V600E^ mutation, as an adjunct diagnostic marker, are routinely screened in the diagnosis of GNT^5–7^.

### The frequency of CD34 expression and BRAF^V600E^ mutation in GNT

As a molecular marker of progenitor cells, CD34 often expresses in low-grade or developmental brain tumors, which are usually accompanied by chronic epilepsy, such as the so-called “low-grade epilepsy-associated neuroepithelial tumors (LEAT)”, but none of normal adult or developing human brain and tumors without epilepsy are CD34 positive^3–5,8,18^. GNT, as the most common tumor type in patients with epilepsy, are also frequently reported with CD34 expression, approximately 50–60%^3,4^. In particular, the ratio of CD34 expression was often higher in GG than DNT^3,5^. For example, Giulioni et al. exclusively observed the CD34 expression in low-grade epilepsy-associated tumors (n = 187), and found 54.1% of CD34 positive in GNT, with a higher expression of CD34 in GG than DNT (74.7% vs. 23.5%)^3^. In our study, we reviewed the CD34 expression in GNT with epilepsy and found 80.2% of tumors were CD34 positive. Also, the ratio of CD34 expression in GG was significantly higher than DNT (87.9% vs. 36.8%; P < 0.001), which was line with the previous studies^3,4,18^.

The BRAF^V600E^ mutations that were primarily found in melanomas also occur in brain tumors, which, similar to CD34, mainly affect low-grade glial or glioneuronal tumors, such as GG, DNT, and PA, as well as pediatric PXA and diffuse astrocytoma^6,7,11^. The mutation of BRAF^V600E^ in GNT was reported ranging from 20 to 60%^6,7,19^. In present study, tumors with BRAF^V600E^ mutation were detected by IHC in 57.8% (59/102) of GNT, including 61.4% of GG, 38.5% of DNT and 50% of mixed GNT, but no difference of BRAF^V600E^ mutation was found between GG and DNT (P = 0.118). Similarly, higher rates of BRAF^V600E^ mutation are often met in GG than DNT, but less statistic differences were recorded between two types of tumors^7,17,18,20^.

In particular, the molecular marker of CD34 expression or BRAF^V600E^ mutation could also be observed in other low-grade tumors with epilepsy, especially in PXA, but less observed in PA, angiocentric glioma (AG), papillary glioneuronal tumor (PGNT) or polymorphous low-grade neuroepithelial tumor of the young (PLNTY)^1–3,5–7^. Interestingly, the new diagnosed entity of PLNTY may share the pathological characteristics with other types of brain tumors, including LEAT, oligodendroglioma, clear cell ependymoma, etc. However, molecular alterations, such as CD34 positive, lack of BRAF^V600E^ mutation, IDH1/2 mutations and 1p19q codeletion, may be helpful to differentiated these types of brain tumors^21^.

### The clinicopathological features of CD34 expression

The relationship between clinicopathology and CD34 expression in GNT has been studied in some studies, but the results were always inconsistent or with limited cases^3–5,19^.

For demographic features, Blümcke et al. found patients with brain tumors with epilepsy and CD34 expression had younger age at seizure onset or at surgery^5^. And Vornetti G, et al. found CD34 expression in LEAT was significantly associated with a longer duration of epilepsy^19^, which was similarly reported by Giulioni et al. who also reported that CD34 expression in LEAT appeared to be significantly related to older age at surgery, higher AED intake, and female sex by univariate analysis^3^. In present study, we found GNT with CD34 expression occurred more in adults than children (OR 2.5, P = 0.014), and CD34 expression was also associated with longer duration of epilepsy (P = 0.027) and drug-resistant epilepsy (P = 0.036) by univariate analysis.

The tumor or pathological characteristics were less reported to be related to GNT with CD34 expression. Lisievici et al. exclusively analyzed the IHC expression pattern of CD34 in GG and found diffuse expression pattern was more in temporal lobe^22^. In present study, we didn’t find the difference of the CD34 expression pattern in GNT, although we found GNT with CD34 expression were more occurring in temporal than non-temporal sites (P = 0.007) by univariate analysis. In particular, tumor-associated FCD was recorded in 26.7% of patients in our surgical cohort, and 5 cases with FCD type I that was not previously diagnosed with tumor-associated FCD in our study were was diagnosed as a new diagnosis of FCD based on the final discussion among two neuropathologists. However, there was no association of tumor-associated FCD with CD34 expression (P = 0.492) or BRAF^V600E^ mutation (P = 0.924) in GNT.

Although CD34 expression tends to occur in lesions with epilepsy, the seizure semiology or EEG finding is seldom reported to be related to GNT with CD34 expression^3,22^. In present study, however, we found patients with CD34 expression in GNT had more tendency of experiencing seizure aura (P = 0.007) and concordant EEG findings (P = 0.032) than those without CD34 expression by univariate analysis.

### The clinicopathological features of BRAF^V600E^ mutation

Although the BRAF^V600E^ somatic mutation in neuronal linage cells (or glial lineage cells) was proved to play a key role in epileptogenic properties (or tumorigenic properties) of GG^23^, the relationships between clinicopathological features and BRAF^V600E^ mutations in GNT were not well studied or with less data^6,16,17,19,20,24^.

Several demographic features were reported to be related to GNT with BRAF^V600E^ mutation, including younger age at surgery for GG (P = 0.005)^24^, younger age of seizure onset for epilepsy-associated brain tumors (P = 0.020)^6^, and female patients for GNT with epilepsy (P = 0.022)^17^. However, Schindler et al. noted no significant differences of patient age at surgery for GG with BRAF^V600E^ mutation^7^; Zhang et al. reported no significant correlation between the BRAF status in GNT and age at surgery, as well as age of seizure onset and duration of epilepsy^17^; and Xing et al. also didn’t find brain tumors with epilepsy and BRAF^V600E^ mutation were associated with gender and duration of epilepsy^6^. In present study, we didn’t find any associations of BRAF^V600E^ mutation in GNT with age of seizure onset, duration of epilepsy and patient gender, except for the older age at surgery (P = 0.015).

For tumor or pathological characteristics, Schindler et al. found GG with BRAF^V600E^ mutation were more in temporal lobe^7^. Prabowo et al. found in both GG and DNT, the presence of BRAF^V600E^ mutation was significantly associated with the expression of CD34^20^. Vornetti et al. found BRAF mutation in LEAT was predominant in right-sided lesions^19^. However, Koelsche et al. found CD34 was not differentially expressed in BRAF wild-type and -mutated tumors of GG^24^, and Xing H, et al. found there was no statistical difference between BRAF^V600E^ mutations and wild type for tumor site^6^. Also, we didn’t find GNT with BRAF^V600E^ mutation were associated CD34 expression, tumor locations, calcification or encystation, et al.

With respect to seizure semiology or EEG findings, BRAF^V600E^-mutated LEAT^19^, as well as GNT^17^, were reported to be with more seizure types. However, we didn’t find GNT with BRAF^V600E^ mutation were associated seizure semiology, except that the concordance of EEG findings was different (discordant vs. concordant, P = 0.031) by univariate analysis.

### Seizure outcomes and tumor recurrence

The correlations between CD34 expression or BRAF^V600E^ mutation in GNT and postoperative seizure outcomes have been evaluated in previous studies^20,25,26^, but they were always with negative results^6,17,19^. For example, Wang et al. found 9 patients with GG had postoperative seizure recurrence, and 8 of them were immunoreactive for CD34^25^, and Prabowo et al. found the expression of BRAF^V600E^ in GNT was associated with a worse postoperative seizure outcome^20^. However, Vornetti et al. didn’t find LEAT with BRAF^V600E^ mutation or CD34 expression were associated with seizure outcomes^19^. Zhang et al. didn’t find any significant correlations between the BRAF status in GNT and postoperative seizure freedom^17^. Also, Xing H, et al. reported there was no statistical difference of epilepsy-associated brain tumors between BRAF^V600E^ mutations and wild type in Engel outcome comparison^6^. Similarly, we defined no differences between CD34 expression (P = 0.807) or BRAF^V600E^ mutation (P = 0.937) in GNT and postoperative seizure outcomes.

The GNT are benign, slow-growing tumors, and patients with GNT rarely experience tumor progression or recurrence, although 5% (or less) of GG (nearly 0% of DNT) with anaplasia or malignant progression were reported in previous studies^1,27^. During the whole follow-up period of 54 months (IQR 26–78 months) in our study, 6 patients had tumor recurrence (GG/5 and DNT/1) and 2 cases of GG had malignant progression, with the 10-year tumor PFS reaching 95%. Through univariate Cox regression analysis, we found the history of GTCS (HR 0.12, P = 0.035), drug-resistant epilepsy (HR 0.13, P = 0.030) and concordant interictal EEG findings (unknown vs. concordant; HR 8.01, P = 0.039) were associated with longer PFS, but only the drug-resistant epilepsy was significant in the multivariate Cox regression analysis. In particular, when compared the Kaplan Meier curves between two groups [tumor with CD34 (+) vs. CD34 (−)] or groups [tumor with BRAF (+) vs. BRAF (−)], no difference was found in patients with detection of CD34 expression or BRAF^V600E^ mutation.

The relationship of CD34 expression or BRAF^V600E^ mutation in GNT with tumor survival (PFS or overall survival) have been studied, previously^16,18,22,27,28^. Although some of studies reported the significant correlation of CD34 expression or BRAF^V600E^ mutation in GNT with tumor recurrence or progression^22^, the extent of the surgical resection (or tumor location), instead of CD34 expression and BRAF^V600E^ mutation, may play an important role of the tumor prognosis of low-grade GNT^16,18,25,27^. However, when analyzing the association of tumor recurrence with resection extent or tumor locations, we didn’t find any statistic differences in resection extent (P = 0.833) and tumor locations (temporal vs. non-temporal, P = 0.498), which may be partly attributed to the high rate of complete tumor resection (99.2%) in our surgical cohort.

## Conclusions

CD34 expression or BRAF^V600E^ mutation in GNT are closely with epilepsy in patients, which may also partly influence the distribution of clinicopathological features of patients. However, CD34 expression or BRAF^V600E^ mutation in GNT may not impact the surgical prognosis of seizure outcome, as well as tumor PFS if complete tumor resection was performed.

## Data Availability

The data used and/or analyzed during the current study are available from the corresponding author on reasonable request.
